# ICF-Based Assessment of Functioning in Daily Clinical Practice. A Promising Direction Toward Patient-Centred Care in Patients With Low Back Pain

**DOI:** 10.3389/fresc.2021.732594

**Published:** 2021-10-26

**Authors:** Charlotte Ibsen, Thomas Maribo, Claus Vinther Nielsen, Mogens Hørder, Berit Schiøttz-Christensen

**Affiliations:** ^1^Department of Public Health, Faculty of Health, Aarhus University, Aarhus, Denmark; ^2^DEFACTUM, Central Denmark Region, Aarhus, Denmark; ^3^Regional Hospital West Jutland, Herning, Denmark; ^4^Department of Public Health, Research Unit of User Perspectives, University of Southern Denmark, Odense, Denmark; ^5^Spine Centre of Southern Denmark, Hospital Lillebaelt, Middelfart, Denmark; ^6^Department of Regional Health Research, University of Southern Denmark, Odense, Denmark

**Keywords:** biopsychosocial approach, international classification of functioning, disability and health, low back pain, patient – centred care, patient-reported outcomes, shared decision-making

## Abstract

**Background:** Patient-centred care has received increased attention in recent years. Patient-Reported Outcomes (PROs) and shared decision-making are key components of Patient-Centred care. Low back pain (LBP) is a complex symptom affected by multiple, interacting factors. Therefore, evidence strongly recommend a biopsychosocial and patient-centred approach in the assessment and management. The International Classification of Functioning, Disability and Health (ICF) provide a biopsychosocial model for describing functioning and disability. ICF is widely acknowledged, but implementation into clinical practice is lacking. To support the use of a biopsychosocial and patient-centred approach in daily clinical practice among patients with LBP we developed a practice-friendly tool based on ICF; the LBP assessment tool.

**Objective:** To compare an ICF-based assessment facilitated by the LBP assessment tool with standard care in terms of the use of PROs and shared decision-making in order to promote patient-centred care in patients with LBP.

**Methods:** A non-randomized controlled design was used. Eligible patients were allocated to one of two groups: the ICF group, assessed with the LBP assessment tool or the control group, assessed with a conventional LBP assessment. Primary outcome includes use of PROs. Secondary outcomes include use of a graphical overview displaying the patient profile and shared decision-making. A patient evaluation questionnaire was used to collect data.

**Results:** Seven hundred ten patients were assessed for eligibility of whom 531 were allocated to the ICF group (*n* = 299) or the control group (*n* = 232). A significantly higher use of PRO data (*p* < 0.00) and the patient profile (*p* < 0.00) was reported in favor of the ICF group. Patients in the ICF group also experienced being more involved in decision-making (*p* = 0.01).

**Conclusions:** This study showed that a functioning assessment, by means of the LBP assessment tool, increased use of PROs and shared decision-making when compared to a conventional LBP assessment. Additionally, this study demonstrated that routine use of ICF-based PRO data and shared decision-making promoted patient-centred care in patients with LBP. The LBP assessment tool may be a strong candidate for a user-friendly ICF-based tool with the potential to support health professionals in a shift toward a biopsychosocial and patient-centred approach to patients with LBP.

## Introduction

Patient-centred care (PCC) has received increased attention in recent years and is now considered a core domain of high-quality healthcare (1). PCC has been defined as “care that is respectful of and responsive to, individual patient preferences, needs and values” (2). It applies a biopsychosocial approach rather than a purely biomedical perspective (3) and it forges a strong partnership between patient and health professional (4, 5). Patient-Reported Outcomes (PROs) (6) and shared decision-making (7) are key components of PCC because they encourage and empower patients to play an active role in their own care. Recently, growing interest in using PRO data directly during the consultation to support management of the individual patient has received widespread attention (8, 9). It turns the focus toward the patient's life experiences, increases awareness to psychosocial problems (10) and can provide new information that may otherwise have been overlooked (11). Besides facilitating clinician-patient communication, PROs may also promote shared decision-making (8). Completion of a PRO prior to a consultation supports patients' self-reflection about their own condition, helps them prioritize issues of importance and identifies topics they wish to discuss during the consultation (12). Additionally, when the PRO data are presented prior to or during the consultation, PROs can increase the awareness of health professionals to patient concerns and prompt health professionals to explore, discuss and address these concerns and subsequently take action (12).

Low back pain (LBP) is a complex symptom affected by multiple, interacting factors such as physical, psychological, social, lifestyle and personal factors (13). The contribution of these factors is unique to each patient (14). To deal with this heterogeneity, a biopsychosocial and patient-centred approach has been recommended to assess and manage LBP, reflecting a holistic approach and emphasizing the importance of active involvement of patients in their own care (13, 15). Despite agreement to apply a biopsychosocial and patient-centred approach, the biomedical approach to managing LBP is still predominant in current clinical practice (16).

Clinicians and researchers use various methods to assess functioning and disability associated with LBP. These methods include clinician-reported outcome (ClinRO) (17) consisting of taking a comprehensive case history and a thorough physical examination and may include the use of PRO data (6). However, commonly used LBP-specific PRO instruments do not cover all domains of the biopsychosocial model (18, 19) like they do not consider factors that are important to patients with LBP (20). As a result, they may not fully capture the experience of individuals with LBP (21). Therefore, developing and using new LBP-specific PRO instruments that are grounded in the biopsychosocial ICF model have been recommended (18, 20, 22). Despite the known advances of using PRO data directly during the consultation to support management of the individual patient, health professionals' use is generally very low (23, 24), and thus also in patients with LBP (9).

The International Classification of Functioning, Disability and Health (ICF) is the internationally-accepted standard for describing and assessing functioning (25). ICF builds not only upon the biopsychosocial model of health and disability, it also provides an exclusive set of categories, which serves as reference units for the standardized reporting of functioning (25). Though, ICF is widely accepted, its implementation into clinical practice is still limited (26). Implementation efforts of ICF include the development of ICF Core Set (27). ICF Core Sets are shortlists of categories selected from the entire ICF classification that are considered essential when assessing the functioning of a person with a specific health condition such as low back pain (28) or in the context of a healthcare or health-related setting, such as in a Rehabilitation setting (29). Though, ICF Core Sets assist the process of defining *what to assess*, ICF categories alone are not operational items and may thus be difficult to assess and use in everyday clinical practice. Consequently, further specification of ICF categories in a user-friendly language is required to promote the use of ICF in daily clinical practice (30–32).

To support the use of a biopsychosocial and patient-centred approach to patients with LBP, we developed an ICF-based assessment tool, the LBP assessment tool (33) to be used in daily clinical practice. The development (33) and field-testing (34) of the tool has previously been published. In brief, the tool was found acceptable by patients and healthcare staff for use in routine clinical practice and it proved to support healthcare staff to apply a more biopsychosocial approach based on the patients perspectives (34). However, the ability of this ICF-based tool to promote patient-centred care has not yet been evaluated. Thus, the objective of this study was to compare an ICF-based assessment facilitated by the LBP assessment tool with standard care in terms of use of PRO data and shared decision-making during the consultation in order to promote patients-centred care in patients with LBP.

## Materials and Methods

### Study Design and Setting

A prospective, non-randomized controlled study was conducted in an out-patient clinic at a secondary-care hospital, the Spine Centre of Southern Denmark. Patients attending the clinical consultation facilitated by the LBP assessment tool (ICF group) were compared with patients attending standard care (control group).

The Spine Centre receives ~12,000 patients with LBP annually. The patients primarily referred from general practice if first-line treatment has not been successful. As standard care all patients attending the Spine Centre receive a multidisciplinary one-time assessment, followed by a plan for rehabilitation (35). Afterwards most patients are referred to outpatient rehabilitation programmes in the primary health sector. Before attending the Spine Centre patients receive an e-mail with a link to a LBP-specific questionnaire, the SpineData PRO. Data from the SpineData PRO are incorporated into the clinical registry SpineData (35). Standard care at the Spine Centre including basic information about the content of the SpineData PRO are described in further detail under the header Control Group.

### Study Population and Allocation

Inclusion criteria for eligible patients were: all patients referred to the Spine Centre with a primary diagnosis of LBP with or without leg pain (sciatica), aged 18–60 years and capable of reading and speaking Danish. The referral team assigned the eligible patients to the ICF group, whereas patients for the control group were identified through the clinical registry SpineData. Allocation was based on time period and patient residence. Patients attending the Spine Centre from November 2017 to April 2018 and living in selected areas of the catchment area were allocated to the ICF group. Patients attending the Spine Centre in August 2018 and living in the remaining parts of the catchment area were allocated to the control group. Thus, the two groups were observed in the same setting but at different periods of time.

### Procedure

Prior to the consultation patients in both groups were asked to complete a PRO instrument at home or at the Spine Centre by using an in-house iPad. During the consultation, healthcare staff completed a clinician-reported outcome (ClinRO) instrument to document the clinical examination. A graphical report displaying data from the PRO and ClinRO instruments was available to the healthcare staff prior to and during the consultation. Data were collected and displayed differently in the two groups (Table 1).

**Table 1:** Collection and presentation of data in the two groups.

	**ICF group**	**Control group**
PRO data collection	PRO-LBP	SpineData PRO
ClinRO data collection	ClinRO-LBP	SpineData ClinRO
Graphical report	Patient profile LBP	SpineData profile

#### ICF Group

**Figure 1:**
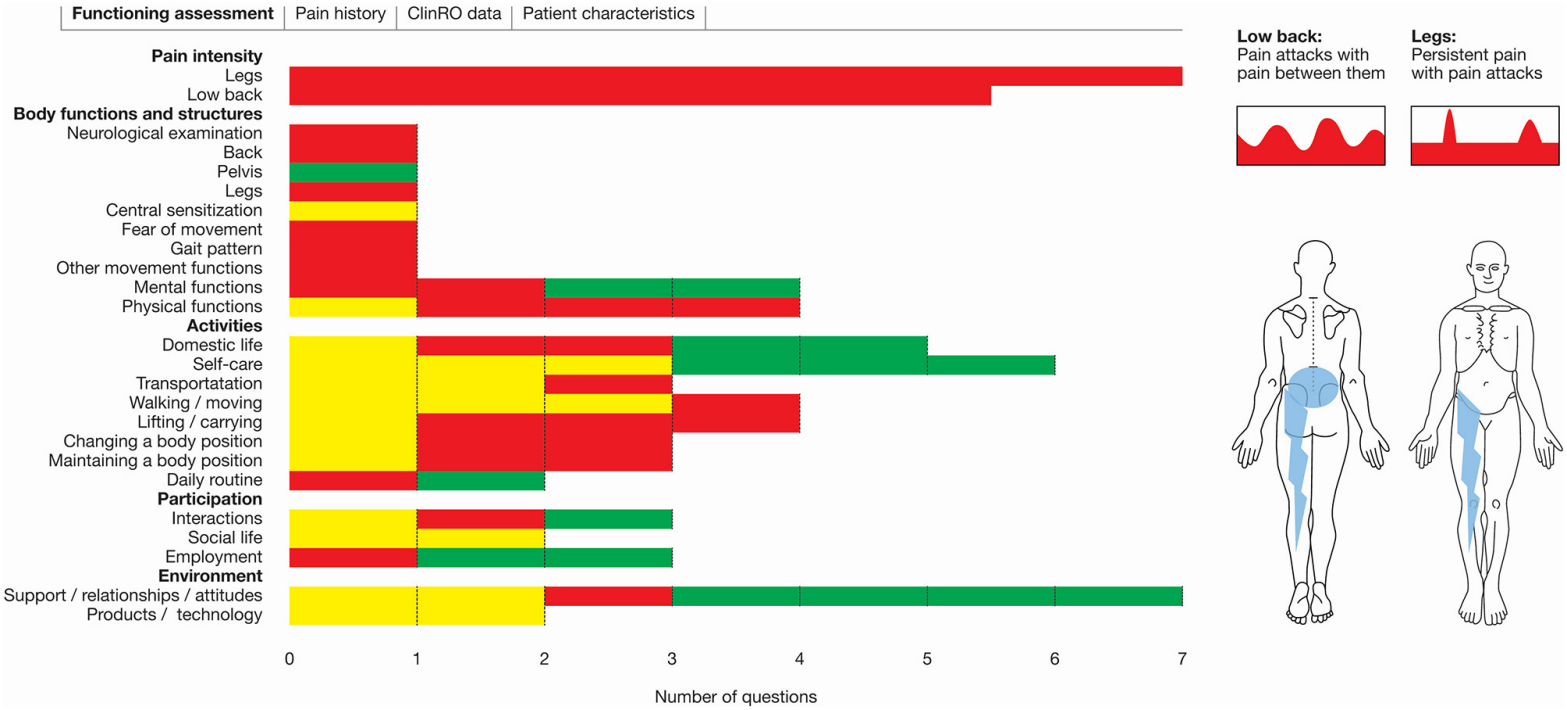
Screenshot patient profile LBP. The ICF components and the corresponding domains are listed together with color-coded bars. Red: Severe disability; Yellow: Mild disability; Green: No disability.

The LBP assessment tool was central in the ICF group. A detailed description of the tool has previous been published (33). In brief the tool was designed to support a biopsychosocial and patient-centred approach to assessment of patients with LBP. It was based on practice-friendly descriptions of ICF categories from the Comprehensive LBP Core Set (28) and the Rehabilitation Set (29). The LBP assessment tool was web-based and build on three features: a PRO instrument (PRO-LBP), a ClinRO instrument (ClinRO-LBP) and a graphical overview (Patient profile LBP). The PRO-LBP (Supplementary Material 1) included information from patients regarding functioning and disability as well as contextual factors. The ClinRO-LBP was designed to assist healthcare staff to standardize the clinical examination. The patient profile LBP integrated data from the PRO-LBP and the ClinRO-LBP by displaying the patient's functioning and disability in a graphical report, structured in accordance with the ICF components; Body functions and structures; activities; participation and environmental factors (Figure 1). The patient profile LBP was designed to be user-friendly and easy to interpret by patients and healthcare staff.

The healthcare staff in the ICF group was *trained* and they practiced a *multidisciplinary teamwork approach* (34). The training comprised an instruction day, a tryout period and a feedback meeting (34). The instruction day focused on how to perform a biopsychosocial assessment of patients within the ICF framework, how to use PRO data and the value of using them during the consultation for individual patient management. Finally, the healthcare staff were instructed in how to use the LBP assessment tool in routine clinical practice (34). The purpose of the tryout period was for healthcare staff to gain confidence in using the LBP assessment tool. The feedback meeting was conducted to discuss observations and share experiences from the tryout period.

*Practicing a multidisciplinary teamwork approach*, is inextricably intertwined with the use of a biopsychosocial approach (36) and is generally associated with a better outcome (37). The use of a multidisciplinary teamwork approach in this study included that the patients in the ICF group underwent an initial clinical examination by a medical doctor or a chiropractor. If the medical doctor or chiropractor needed supplementary assessment to be able to achieve a full understanding of the patient's situation or to decide on the rehabilitation plan, an extended LBP assessment was performed by a physiotherapist. All patients consulted a nurse regarding medicine and everyday life issues. To support the multidisciplinary team approach, the healthcare staff was allowed to work collaboratively when assessing, planning, and evaluating the patient, just like they had a team meeting each day to share expertise and knowledge and discuss their patients. The multidisciplinary team in the ICF group comprised a selected group of healthcare staff from the Spine Centre (*n* = 7) with specialist expertise (knowledge and skills) in managing patients with LBP (Table 2).

**Table 2:** Healthcare staff in the ICF group.

**Gender**	**Age**	**Background**	**Years working with LBP**	**Years working at the spine centre**
M	51	Chiropractor	25	8
F	63	Medical doctor, Social medicine	9	7
F	45	Nurse	8	8
F	50	Nurse	6	6
F	57	Medical doctor, Rheumatologist	20	4
F	38	Physiotherapist	12	7
F	41	Physiotherapist	15	8

Patients in the ICF group received an e-mail with information about the project, an informed consent form and an electronic link to the PRO-LBP. Patients' PRO data were available to healthcare staff (Figure 1) and designed to be used in the preparation of and during the consultation.

#### Control Group

**Figure 2:**
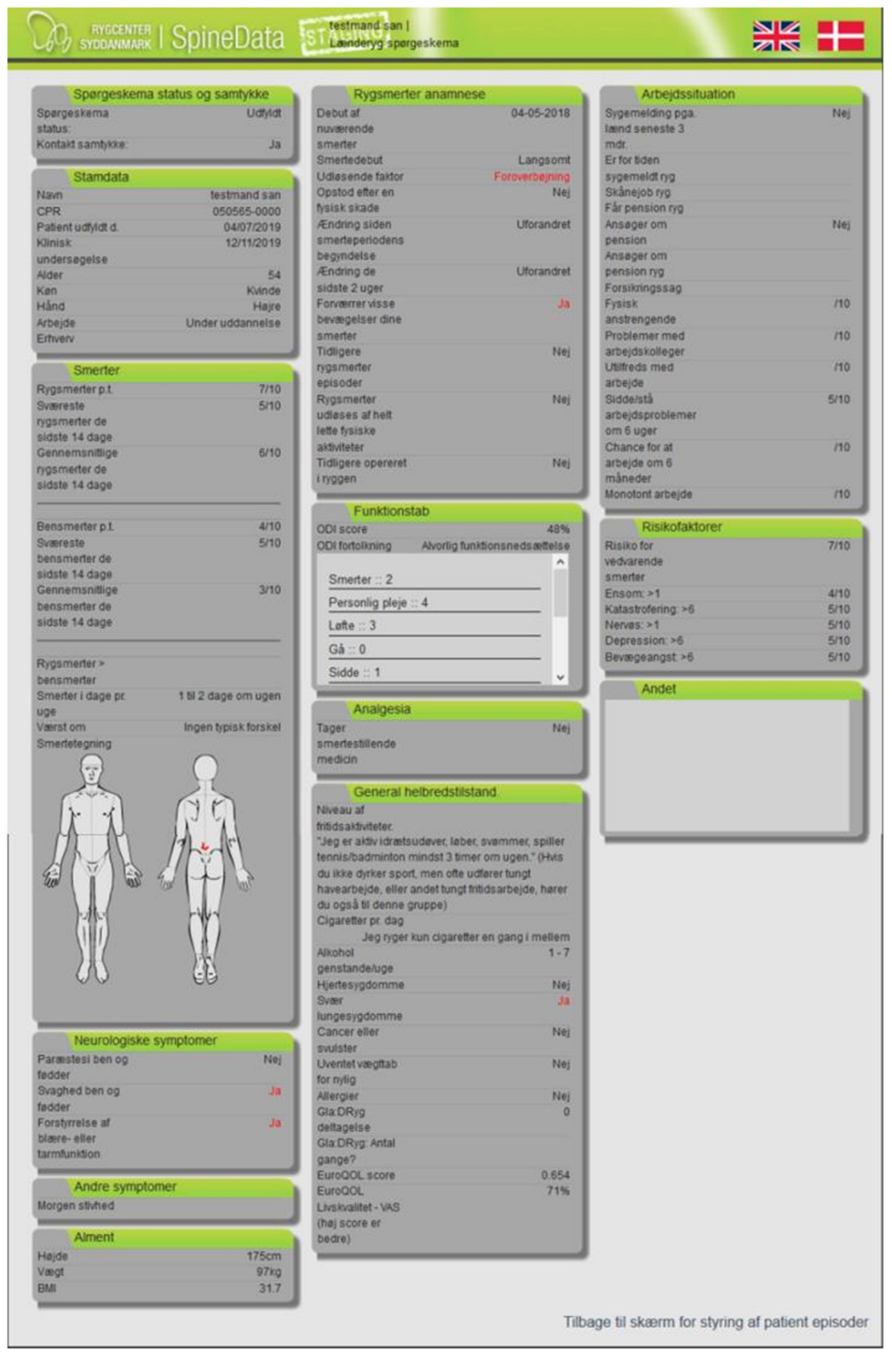
Screenshot SpineData profile.

The control group followed standard care at the Spine Centre where data from the clinical registry SpineData were used. The SpineData encompassed the SpineData PRO, SpineData ClinRO and summary reports of data (35). The SpineData PRO comprised a combination of established PRO instruments, such as the 23-item Roland Morris Disability Questionnaire (RMDQ) (38) and EuroQol (39). Overall, the SpineData PRO comprised items about health domains: pain, activity limitation, participation, mental functions, physical impairment and contextual factors. Data from the SpineData PRO were incorporated into the clinical registry SpineData, including ClinRO data (35). Summary reports were generated for staff, and staff could access these reports from the individual patient's SpineData profile before seeing a patient for the initial consultation (Figure 2). Staff was continuously trained to use SpineData.

Patients in the control group received an electronic link to the SpineData PRO (35) before the consultation. They underwent a conventional LBP clinical examination performed by a multidisciplinary team with extensive experience in managing patients with LBP.

#### Outcome Measures

The primary outcome was use of patients PRO data during the consultation. The secondary outcomes included presentation of the graphical overview showing patient's profile (Figures 1, 2) and shared decision-making.

The use of patients PRO data (Table 3, item no 1) and their patient profile (Table 3, item no 2) during the consultation was measured with two self-constructed items (Table 3). Item no 1 was designed to assess to which degree patients' PRO data was used in the dialogue with the health professional, and rated from 1 (not at all) to 5 (very much). This response option was adapted from the Patient-Reported Outcomes Measurement Information System (PROMIS®) (40, 41). Item no 2 was designed to assess whether the patients saw their own patient profile during the consultation. A nominal scale was used (Yes/No). Shared decision-making was measured with the 9-item Shared Decision-Making Questionnaire (SDM-Q-9), designed to measure the extent to which patients are involved in shared decision-making (42). The SDM-Q-9 consist of nine statements, which can be rated on a 6-point Likert scale ranging from 0 (completely disagree) to 5 (completely agree), with a raw total score between 0 and 45 points. A high score indicates a high patient experience of shared decision-making. The SDM-Q-9 has shown good psychometric properties in a Danish setting (43). A patient evaluation questionnaire was constructed to measure primary and secondary outcomes, comprising the self-constructed items and the SDM-Q-9 (Supplementary Material 2).

**Table 3:** Self-constructed items to measure use of PRO and patient profile.

**Items**	**Response options**
1. To which degree was your responses from the PRO used in your dialogue with the health professional?	□ Not at all (1) □ Very little (2) □ Somewhat (3) □ Quite a lot (4) □ Very much (5)
2. Did you see this report during the consultation? [a screenshot were presented to the patient]	□ Yes □ No

#### Data Collection

Patients received a link to the patient evaluation questionnaire immediately after their consultation. Data were obtained through SurveyXact®, and non-responders received up to three reminders. Baseline characteristics regarding patients in the ICF group were collected using the PRO-LBP and regarding patients in the control group the SpineData PRO were used. Consequently, pain intensity and disability were measured with two different instruments. A visual analog scale (VAS 0-100) and the Oswestry Disability Index (ODI) were included in the PRO-LBP (ICF group) because the new PRO-LBP aimed to standardize the use of instruments across medical and surgical specialties at the Spine Centre. Therefore, VAS and ODI were used in the ICF group. Patients in the control group completed the SpineData PRO, which included a numeric rating scale (NRS 0-10) and the 23-item RMDQ. These two instruments were applied as they were standard instruments in the SpineData registry at the time of the study. NRS 0-10 and 23-item RMDQ has been used at the Spine Centre since 2011, as part of standard care.

#### Patient and Public Involvement

Patients experiencing LBP and staff from the Spine Centre were involved in the design of the LBP assessment tool (33). Their contributions further qualified the tool and identified elements of importance for its use in routine clinical practice.

### Statistical Analysis

Descriptive statistics were used to describe the patients. To compare patients' pain intensity between groups, NRS data (0–10) were converted into a VAS (0–100). To be able to compare scores between the ODI and the RMDQ we had to divide the RMDQ sum scores into subgroups of disability (44). Data regarding “use of PRO data” were collected on a five-point scale from 1 to 5 to allow for differentiation. During analysis the variable were dichotomized (0 = no; 1 = yes) by collapsing the response options 1 and 2 into 0, which corresponds to “no,” and the response options 3, 4 and 5 into 1, which corresponds to “yes.” Dichotomization was performed because it was estimated to be more comparable to clinical practice. Categorical variables were analyzed using a Chi-square test. The raw score of the SDM-Q-9 was transformed into a 0–100 scale, by multiplying the raw score by 20/9 (42). The 0–100 scale is intuitively interpretable, and the authors of the SDM-Q-9 encourage the use of the transformed scale (42). The Wilcoxon rank-sum test was used for analysis. A non-responder analysis was performed on age and gender. An explorative analysis was conducted to investigate whether the potential differences in patient characteristics had an interacting impact on the use of PRO and shared decision-making. Age and gender were added to the explorative analysis. The level of statistical significance was set at a *p*-value < 0.05. STATA version 16 was used for all analyses.

### Ethics

The study was approved by the Danish Data Protection Agency (file no. 1-16-02-477-16) and the Danish Patient Safety Authority (file no. 3-3013-2513-1). According to the Central Denmark Region Committees on Health Research Ethics, ethical approval was not required (file.no. 150/2016). All patients and healthcare staff received oral and written information about the study, and written consent was obtained before participation.

## Results

**Figure 3:**
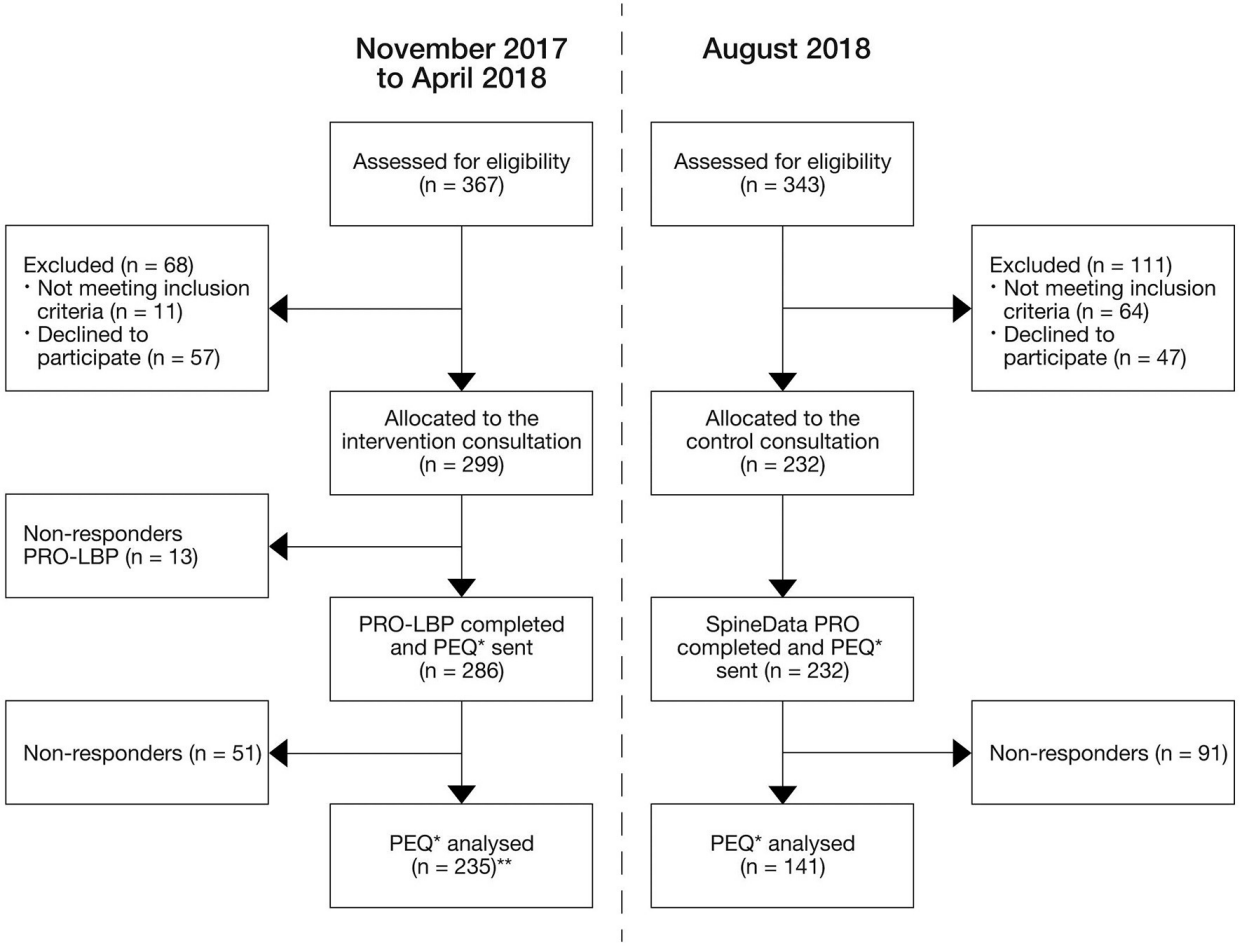
Study flowchart. *PEQ, Patient evaluation questionnaire; **Data regarding the item “use of the patient profile LBP in the consultation” were missed in 4 patients due to technical issues, thus analysis were based on 231 patients.

Between November 2017 and April 2018, 299 patients were allocated to the ICF group. In August 2018, 232 patients were allocated to the control group (Figure 3).

In total, 235 patients from the ICF group completed the patient evaluation questionnaire (response rate: 82%); this applied to 141 in the control group (response rate: 61%). Characteristics of patients who completed the questionnaire are presented in Table 4.

**Table 4:** Demographics and clinical characteristics of study participants.

	**ICF group**	**Control group**
	**(*n* = 235)**	**(*n* = 141)**
**Patient characteristics**		
Gender, women, *n* (%)	113 (48)	84 (60)
Age, years, mean (SD)	45 (11)	46 (11)
**Disability, mean (SD)**		
Oswestry Disability Index (0–100%)	34 (16)	–
Roland Morris Disability Questionnaire (0–23)	–	14 (6)^a^
**Pain duration** **>** **3 months**, ***n*** **(%)**		
Back pain	197 (90%)^b^	110 (81%)^c^
Leg pain	142 (75%)^d^	98 (80%)^e^
**Pain intensity, mean (SD) (0–100)**		
Back pain	70 (21)^b^	80 (20)^f^
Leg pain	67 (23)^d^	70 (30)^c^
On sick leave (full- or part-time), *n* (%)	71 (32)^g^	54 (48)^h^
Current smoker, *n* (%)	53 (23)	39 (29)^i^
Previous back surgery, *n* (%)	26 (11)	26 (19)^c^
Comorbidity in addition to back pain, *n* (%)	99 (42)	52 (38)^c^
General health, EQ-5D VAS, mean (SD)	53 (23)	47 (25)^i^

a *n = 133;*

b *n = 220;*

c *n = 136;*

d *n = 188;*

e *n = 123;*

f *n = 138;*

g *n = 225;*

h *n = 112;*

i *n = 135*.

All participating patients reported moderate disability, corresponding to an ODI score of 34% (21–40%: moderate disability) for the ICF group and an RMDQ sum score of 14 (13–18: moderate disability) for the control group (Table 4). We found some statistically significantly differences in patient characteristics between the two groups. Patients in the ICF group reported having a longer back pain duration (*p* = 0.02) and a better general health (*p* = 0.01) than the control group. On the other hand, the control group had a higher proportion of sick leave (*p* < 0.00) and previous surgery (*p* = 0.03) than the ICF group.

A non-responder analysis was performed on 51 patients from the ICF group (18%) and 91 patients from the control group (39%). No significant differences were found in gender. However, in both groups, the non-responders were significantly older than the responders. Non-responders were 3.7 (95% CI: 0.5; 7.02) years older in ICF group (*p* = 0.03) and 6.7 (95% CI: 3.9; 9.6) years older in control group.

### Outcomes

When use of patients PRO data during the consultation was compared, 78% (95% CI: 72; 82) of patients in the ICF group reported that their PRO data were used compared to 58% (95% CI: 49; 65) of patients in the control group (*p* < 0.00). Use of patient's profile and shared decision-making was significantly higher in the ICF group compared with the control group (Table 5).

**Table 5:** Comparison; use of PRO data, patient's profile and shared decision-making.

	**ICF group (*n* = 235)**	**Control group (*n* = 141)**	
	**Value**	**Value**	***p*-value**
**Primary outcome**			
Use of PRO data^*^	78% (72; 82)	58% (49; 65)	*P* <0.00
**Secondary outcomes**			
Use of patient's profile^*^	68% (61; 73)^#^	43% (35; 52)	*P* <0.00
Shared decision-making^†^	71 (68; 73)	66 (62; 69)	*P* = 0.01

*PRO data, Patient-reported Outcome data;*

* *Data are presented as percentages with 95% confidence intervals (CI);*

† *Data are presented as sum scores 0–100 with 95% confidence intervals;*

# *n = 231*.

Explorative analysis regarding differences in patients' characteristics between groups revealed that the higher proportion of patients on sick leave in the control group was the only parameter associated with the use of PRO data, as patients on sick leave more frequently reported that their PRO data were not used during the consultation (*p* = 0.02). Additional explorative analysis revealed that sick leave was not associated with the use of PRO data for the total group of patients (*p* = 0.06). Furthermore, no association was found between sick leave and shared decision-making (*p* = 0.85).

## Discussion

We found that use of PRO data and patients' experiences of being involved in decision-making was significantly higher in the ICF group compared with the control group. Thus, the LBP assessment tool encouraged healthcare staff to discuss patients' concerns and facilitated active engagement of patients during the consultation compared with patients receiving standard care. Overall, this study showed that use of this ICF-based tool had a high impact on the consultation process, as patients in the ICF group experienced a more patient-centred consultation (higher reported use of PRO data and patient profile, and higher shared decision-making than in the control group).

The LBP assessment tool was based on the original ICF category definition from Core Sets presented in a user-friendly language with the potential to facilitate the utility of ICF in routine clinical practice. China (31), Italy (32) and recently Japan (30), have taken the lead in developing 'simple intuitive descriptions' of ICF categories to inform a system-wide implementation of ICF in routine clinical practice. With the development of the LBP assessment tool, we have laid a solid foundation and starting point for a process in Denmark toward generating 'simple intuitive descriptions' of ICF categories contained in the LBP Core Set and the Rehabilitation set. This may be the first small step toward a system-wide implementation of ICF in Denmark among patients with LBP.

Our results showed that patients in the ICF group reported a significantly higher use of their PRO data during the consultation (78%) compared with the control group (58%). In two previous studies, patients and healthcare staff found that use of PRO data should reach a level of around 80–85% to be feasible and acceptable (45, 46). In the ICF group, we were close to this level, but we were far from an acceptable level in the control group. The reduced use of PRO data in the control group was disturbing because the SpineData PRO has been used in the Spine Centre since 2011 (35). However, this emphasizes that the routine use of PRO data during the consultations is challenging and needs persistent facilitation (47) and training of healthcare staff (48, 49). An essential step in the development of the LBP assessment tool was an interview with the healthcare staff from the Spine Centre (33). The interview revealed that their use of the SpineData PRO varied considerably, mainly because the items did not reflect patients' everyday life and because the staff did not find the SpineData to be beneficial for routine clinical practice (33). This supports that acceptance by healthcare staff is crucial to the success of using PRO data during the consultation (50, 51). In a previous field-testing, we found that the LBP assessment tool gave a smooth and positive consultation based on the patient perspective because patients found it easy to fill out the PRO-LBP and their responses were useful to the healthcare staff (34). This was supported by the results of the present study as the 20% higher use of PRO data in favor of the ICF group may indicate that the healthcare staff accepted the PRO-LBP. Additionally, the healthcare staff appreciated the structured presentation of the PRO data in accordance with the ICF components, and they found the items meaningful and relevant. With the LBP assessment tool, the healthcare staff addressed the patient's concerns and discussed these within the clinical agenda, resulting in patients feeling more involved in the consultation process.

Patients in the ICF group exhibited a significantly higher degree of shared decision-making than the control group. This result supported that the LBP assessment tool facilitated clinician-patient communication, thus promoting patients' experience of shared decision-making. These results are in accordance with previous research (8, 46, 52, 53). Nonetheless, our results should be interpreted with caution, as the difference of 5 points in a 0–100 sum score may be smaller than the measurement error of the questionnaire. However, measurement error and minimal important change values are unknown for the SDM-Q-9 (54). Thus, measurement error and minimal important change values should be determined in future studies to inform the interpretation of SDM-Q-9.

Integrating PRO data in the clinical consultation is a challenging process, and several issues need to be considered carefully before the implementation (8, 47, 55). These include involvement of patients and healthcare staff in as many steps as possible (47, 55, 56), training of healthcare staff (48, 49), appointment of a facilitator operating in the local setting (47) and ensuring that data are acceptable and meaningful to both patients and healthcare staff (55, 57). To address these factors in the development of the LBP assessment tool, we carried out several steps (33, 34, 58). Firstly, we interviewed the patients (58) and healthcare staff to explore their needs and ensure comprehension and clinical meaningfulness of the items (33). Secondly, we trained healthcare staff to promote ownership and correct use of the PRO data (34). Thirdly, we appointed a facilitator to work with the multidisciplinary team and adapt the LBP assessment tool to the local context (34). Fourthly, we conducted a field-testing and found the LBP assessment tool meaningful and acceptable to both patients and healthcare staff (34). Finally, we developed the patient profile LBP (Figure 1) to position PRO data in the consultation and facilitate active use of PRO data, as previously requested by patients treated at the Spine Centre (58). The above has been shown to be a precondition to integrate PRO data into routine clinical practice (6). We believe that the systematic and comprehensive development process including involvement of patients and healthcare staff, specific training of staff and a feedback meeting are major strengths of the development and field-testing of the LBP assessment tool. All of these elements together may explain the high use of PRO data and shared decision-making in the ICF group. However, further studies are needed to achieve a better understanding of these elements and to determine whether the impact of the LBP assessment tool can be attributed to organizational structure or the training of the healthcare staff. Furthermore, the high use of the LBP assessment tool indicated that patients and healthcare staff found it meaningful because it supported the consultation process.

This study has some limitations that need to be recognized. Firstly, we used a non-randomized study design because it was the most applicable design to be implemented at the Spine Centre due to organizational changes during the planning and completion of the study. Consequently, the allocation of patients was based on a non-randomized selection, and we thus we may have introduced selection bias. Differences in patient characteristics between groups were found. However, the direction of these characteristics was mixed. On the one hand patients in the ICF group experience having a longer back pain duration and a better general health; on the other hand, the control group had a higher proportion of sick leave and previous surgery than the ICF group. Due to the mixed direction of patient characteristics, it is unclear whether differences in patients' characteristics has affected our result in favor of the ICF group. Adjusting for imbalances in patient characteristics were considered, but because some health professionals could have seen patients in both groups the assumption of independence between data was not meet. Therefore, analysis adjusting for imbalance in patient characteristics was not performed. In addition, explorative analysis revealed that the higher proportion of patients on sick leave in the control group was the only parameter associated with the use of PRO data. To determine whether sick leave could have modified the observed effect of the LBP assessment tool, we tested if there was an association between sick leave and use of PRO data in the total population, and between sick leave and shared decision-making. No associations were found which reduced the risk of selection bias and supported the effect of the LBP assessment tool. It is also worth to mention that we used different instruments to measure disability and pain in the two groups. It is unclear whether this has affected our results. Furthermore, the beneficial effect of the LBP assessment tool was tested in a “real-world” setting, thereby increasing external validity on the one hand and decreasing internal validity on the other. Secondly, although the patient evaluation questionnaire was short, sent immediately after the consultation and its content was considered relevant for patients, the response rate in the control group was rather low (61%). A low response rate may introduce bias and affect the validity of a study (59). However in accordance to survey research, a response rate of at least 60 % is considered sufficient to ensure that non-response bias threatens the validity of the findings (60). Non-response bias may be an issue when differences exist between responders and non-responders (61). In our study, we found an age difference between responders and non-responders. Overall, the study participants were rather young (mean age of 44 years), and the minor age differences were probably not of critical importance to the outcome. Besides age and gender, we were not allowed to collect additional patient characteristics on the non-responders due to the general data protection regulation (62). Thirdly, missing information regarding patients' educational level may have introduced confounding, because educational level could be associated with patient involvement. In general, highly educated patients opt for greater involvement than less educated patients (63–65). Moreover, highly educated patients tend to have a greater capacity for attaining and understanding basic health information needed to make appropriate health decisions (63). If we assume that patients in the ICF group had a higher educational level than patients in the control group, this might have led to an overestimation of the effect of the LBP assessment tool. However, to properly understand if educational level could be a potential confounder, these data need to be collected and analyzed in future research. Fourthly, a potential bias of this study was that we may have introduced a type 1 error due to the pre-specified level of significance (α = 0.05). Nevertheless, the *p*-values connected to the primary and secondary outcomes were less than the pre-specified *p*-value, which underpins a rejection of no difference and a consolidation of the effect of the LBP assessment tool. Finally, the risk of contamination between the ICF group and the control group must be considered. The two groups were observed in two different time periods, with patients in the ICF group being observed before patients in the control group. Healthcare staff assessing patients in the ICF group may also have assessed patients in the control group. Thus, they could have passed on their skills and experiences from the ICF group into the control group in such a way that their behavior changed when they assessed patients in the control group. However, the significantly lower use of PRO data and shared decision-making in the control group may be an indication that contamination was not a problem and thus unlikely to have affected our results.

## Conclusion

This study showed that an ICF-based functioning assessment, by means of the LBP assessment tool, increased use of PROs and shared decision-making when compared to a conventional LBP assessment. Additionally, this study demonstrated that routine use of ICF-based PRO data and shared decision-making promoted PCC in patients with LBP, being the key components of PCC. The LBP assessment tool may be a strong candidate for a user-friendly ICF-based tool with the potential to support health professionals in a shift toward a biopsychosocial and patient-centred approach to patients with LBP. Given the significant impact of the LBP assessment tool on the use of PRO data and shared decision-making, further research to determine whether this impact was attributed to organizational structure or the training of the healthcare staff is important. In addition, more studies are warranted to investigate whether the LBP assessment tool can be used in other LBP settings such as primary care which is where the majority of patients with LBP are managed.

## Data Availability Statement

The datasets generated and/or analysed during the current study are not publicly available due to individual privacy which could be compromised, but are available from the corresponding author on reasonable request.

## Ethics Statement

The study was approved by the Danish Data Protection Agency [file no. 1-16-02-477-16] and the Danish Patient Safety Authority [file no. 3-3013-2513-1]. Ethical review and approval was not required for the study on human participants in accordance with the local legislation and institutional requirements [file no. 150/2016]. The patients/participants provided their written informed consent to participate in this study.

## Author Contributions

CI, BSC, TM, CVN, and MH designed and planned the study in collaboration. CI carried out the study, performed the analysis, and wrote the first draft of the manuscript. CI, BSC, TM, CVN, and MH discussed the results and contributed to the final manuscript. All authors contributed to the article and approved the submitted version.

## Funding

The study was supported by Aarhus Universitet, Grant/Award Number: 493.000 DKK; Danish Health Authority, Grant/Award Number: 83.661 DKK; Health Research Fund of Central Denmark Region, Grant/Award Number: 439.445 DKK; Spine Centre of Southern Denmark, Grant/Award Number: 500.000 DKK; DEFACTUM, Central Denmark Region, Grant/Award Number: 800.000 DKK; The Danish Rheumatism Association, Grant/Award Number: 60.000 DKK.

## Conflict of Interest

The authors declare that the research was conducted in the absence of any commercial or financial relationships that could be construed as a potential conflict of interest.

## Publisher's Note

All claims expressed in this article are solely those of the authors and do not necessarily represent those of their affiliated organizations, or those of the publisher, the editors and the reviewers. Any product that may be evaluated in this article, or claim that may be made by its manufacturer, is not guaranteed or endorsed by the publisher.
