# Supplementary material for: ICF-Based Assessment of Functioning in Daily Clinical Practice. A Promising Direction Toward Patient-Centred Care in Patients With Low Back Pain
Source: Front Rehabil Sci. 2021 Oct 26;2:732594. doi: 10.3389/fresc.2021.732594 (PMC9397761; doi:10.3389/fresc.2021.732594)
Supplement: Supplementary file 1 [file Table_1.DOCX]

Supplementary Material

**Supplementary Material 1: PRO-LBP**

| ICF code | Item text | Response options |
| --- | --- | --- |
| **1. Patient information** | | |
| PF | Please specify your height in cm | Open text box |
| PF | Please specify your weight in kg | Open text box |
| PF | Do you smoke | Yes  No, but I am a former smoker  No, I have never smoked |
| PF | How many units of alcohol do you drink a week | 0 units a week 1-7 units a week 8-14 units a week More than 14 units a week |
| PF | Have you had surgery in the back before | No Yes, once Yes, twice Yes, three or more times |
| PF | Specify if you suffer from or have suffered from one or more of the following diseases/symptoms | Heart disease Lung disease Disease in the brain- /nervous-system Diabetes Cancer Unintended weight loss Other disease that affects your  walking Other disease that causes pain Allergies Use of corticosteroids tablets for more than 3 months |
| **2. Pain** | | |
| **Pain location** | | |
| b280 | Where is current pain/symptoms located  [*patients are shown pictures*] | Low back  Low back with radiating pain to one   or both legs  Legs |
| b28015 | You have marked having leg-pain in one or both legs – where is your leg-pain located  [*patients are shown pictures*] | Back side  Front side |
| b28015 | You have marked having pain in lower back with radiating pain to one or both legs –  Mark the picture that best indicates your current leg-pain  [*patients are shown pictures*] | Right leg: Radiation to thigh Radiation to calf Radiation to foot Left leg: Radiation to thigh Radiation to calf Radiation to foot |
| b28015 | You have marked having pain to one or both legs –  Mark the picture that best indicates your current leg-pain  [*patients are shown pictures*] | Right leg: Radiation to thigh Radiation to calf Radiation to foot Left leg: Radiation to thigh Radiation to calf Radiation to foot |
| b28015 | Pain on the front side of the leg with/without pain in low back:  Mark the picture that best indicates your current pain  [*patients are shown pictures*] | Right leg: Radiation to thigh Radiation to calf Radiation to foot Left leg: Radiation to thigh Radiation to calf Radiation to foot |
| **Back pain** | | |
| b28013 | How was the onset of your back pain | Gradual  Sudden |
| b28013 | What activated your back pain | Unknown cause Lifting (heavy or light) Bending over A sudden movement |
| b28013 | Since this current episode of back pain your back pain has become... | Worse  Unchanged  Better |
| b28013 | Place a mark on the line to show the strongest pain you have experienced within the past 7 days | 0-100 |
| b28013 | Place a mark on the line to show the weakest pain you have experienced within the past 7 days |  |
| b28013 | Mark the picture that best describes the course of your back pain [*patients are shown the pictures from painDETECT]* | Persistent pain with slight   fluctuations  Persistent pain with pain attacks  Pain attacks without pain between   them  Pain attacks with pain between them |
| b28013 | At what time of the day is your back pain the worst | No difference In the morning In the morning and evening Worsens during the day In the evening At night |
| b28013 | What can help reduce your back pain | Movement, e.g. going for a walk Lying down Sitting something else Nothing |
| b28013 | What can increase your back pain |  |
| b28013 | For how long have back pain been a problem for you | More than 5 years 1-5 years 6-12 months 3-6 months 1-3 months Less than one month |
| **Leg-pain** | | |
| b28015 | How was the onset of your leg-pain | Gradual  Sudden |
| b28015 | What activated your leg-pain | Unknown cause Lifting (heavy or light) Bending over A sudden movement |
| b28015 | Since this current episode of leg-pain your leg-pain has become... | Worse  Unchanged  Better |
| b28015 | Place a mark on the line to show the strongest pain you have experienced within the past 7 days | 0-100 |
| b28015 | Place a mark on the line to show the weakest pain you have experienced within the past 7 days |  |
| b28015 | Mark the picture that best describes the course of your back pain  [*patients are shown the pictures from painDETECT]* | Persistent pain with slight   fluctuations  Persistent pain with pain attacks  Pain attacks without pain between   them  Pain attacks with pain between them |
| b28013 | At what time of the day is your leg-pain the worst | No difference In the morning In the morning and evening Worsens during the day In the evening At night |
| b28013 | What can help reduce your leg-pain | Movement, e.g. going for a walk Lying down Sitting something else Nothing |
| b28013 | What can increase your leg-pain |  |
| b28013 | Do you suffer from the following pain/symptoms in your legs/feet | Burning sensations Tingling or prickling sensations Numbness  Weakness Paralysis |
| b28013 | For how long have leg-pain been a problem for you | More than 5 years 1-5 years 6-12 months 3-6 months 1-3 months Less than one month |
| **Pain relieving drugs** | | |
| e1101 | How often do you use pain-relieving drugs for your back- and/or leg-pain | Daily  Several times a week  Never |
| e1101 | What kind of pain-relieving drugs do you use | Paracetamol  NSAIDs  Tramadol  Gabapentin  Lyrica  Chloroxazaxone  Amitriptyline |
| **Pain in other body parts** | | |
| b280 | Have you within the last 7 days had pain in other body parts than back and legs | No  Yes, write here: _________ |
| **3. Mobility** | | |
| d4153 | Are you able to stay in a seated position for some time as required | Without any difficulty  With a little difficulty  With some difficulty  With much difficulty  Unable to |
| d4150 | Are you able to stay in a lying position for some time as required |  |
| d4154 | Are you able to stand up for as long as necessary |  |
| d4103 | Are you able to get into and out of a seated position and changing body position from sitting down to any other position |  |
| d4100 | Are you able to get into and out of a lying down position or changing body position, from horizontal to any other position |  |
| d4105 | Are you able to tilt the back downwards or to the side such as in bowing or reaching down for an object |  |
| d430 | Are you able to lift or carry an object from one place to another |  |
| d4450 | Are you able to pull a heavy object (5 kg) towards you |  |
| d4451 | Are you able to open a heavy door by pushing |  |
| d4452 | Are you able to reach for something |  |
| d450 | Are you able to walk |  |
| d4551 | Are you able to climb stairs |  |
| d4552 | Are you able to run |  |
| d460 | Are you able to move around inside and outside your home |  |
| d4700 | Are you able to be a passenger in a car or take the bus |  |
| d4751 | Are you able to drive a car |  |
| d4750 | Are you able to ride a bicycle |  |
| **4. Self-care** | | |
| d540 | Are you able to put on and take off clothes | Without any difficulty  With a little difficulty  With some difficulty  With much difficulty  Unable to |
| d5101 | Are you able to take a shower |  |
| d520 | Are you able to carry out personal care, such as looking after skin, teeth, hair and nails |  |
| d530 | Are you able to go to the toilet on your own |  |
| d550 | Are you able to have a meal |  |
| d570 | Are you able to look after your own health, e.g. to maintain a balanced diet and an appropriate level of physical activity |  |
| **5. Domestic life** | | |
| d640 | Are you able to manage a household such as cleaning or washing clothes | Without any difficulty  With a little difficulty  With some difficulty  With much difficulty  Unable to |
| d630 | Are you able to prepare a meal |  |
| d6200 | Are you able to go shopping |  |
| d650 | Are you able to maintain and repair households and other personal objects indoor and outdoor |  |
| d660 | Are you able to assist family and friends with domestic life |  |
| **6. Work and employment** | | |
| d850 | State your current employment status | Employed Flexiijob Stay-at-home husband/wife  Unemployed Enrolled in education  Vocational training  Disability pension Age-related pension |
| nd | Are you currently on sick leave due to pain/symptoms in your back and/or legs | No  Yes, full-time  Yes, part-time |
| d845 | Are you able to seek employment, e.g. write an application or attend a job interview | Without any difficulty  With a little difficulty  With some difficulty  With much difficulty  Unable to |
| d859 | Are you able to have other kinds of jobs or employment |  |
| **7. Community, social and civic life** | | |
| d910 | Are you able to engage in community social life, e.g. being engaged in a club or an organization | Without any difficulty  With a little difficulty  With some difficulty  With much difficulty  Unable to |
| d920 | Are you able to participate in any form of play, recreational or leisure activity, e.g. games, sports, exercise programs, go to the movies, or travel |  |
| **8. Interpersonal interactions and relations** | | |
| d760 | Does your back-/leg pain now limit your relation to your family | Not at all  Very little  Somewhat  Quite a lot  Very much |
| d710 | Does your back-/leg pain now limit your interaction with other people, e.g. showing consideration and esteem when appropriate, showing respect |  |
| d770 | Does your back-/leg pain now limit you in being in a relationship |  |
| **9. General tasks and demands** | | |
| d230 | Does your back-/leg pain now limit you in budgeting time and making plans for activities during the day | Not at all  Very little  Somewhat  Quite a lot  Very much |
| d240 | Does your back-/leg pain now limit you in dealing with situations involving stress and distraction, e.g. driving a car during heavy traffic or taking care of many children |  |
| **10. Physical functions** | | |
| b455 | Does your back-/leg pain now limit your physical endurance | Not at all  Very little  Somewhat  Quite a lot  Very much |
| b6200 | Does your back-/leg pain now limit your urination |  |
| b525 | Does your back-/leg pain now limit your defecation functions |  |
| b640 | Does your back-/leg pain now limit your sexual functions |  |
| **11. Mental functions** | | |
| b1300 | Does your back-/leg pain now limit your energy level | Not at all  Very little  Somewhat  Quite a lot  Very much |
| b134 | Does your back-/leg pain now limit your sleep |  |
| b152 | Does your back-/leg pain now affect your emotional functions, e.g. being worried, sad or irritable |  |
| b126 | Does your back-/leg pain now affect your  temperament and personality functions, e.g. problems acting inappropriately in different situations |  |
| **12. Products and technology** | | |
| e1550 | Does your back-/leg pain now limit you in getting around inside because of narrow doors, high doorsteps, stairs, carpets etc. | Not at all  Very little  Somewhat  Quite a lot  Very much |
| e1351 | Does your back-/leg-pain cause need of assistive products and technology at your workplace, e.g. adjustable table |  |
| **13. Support and relations** | | |
| e310 | Do you get sufficient support from your immediate family regarding your back-/leg-pain | Not at all  Very little  Somewhat  Quite a lot  Very much |
| e355 | Do you get sufficient support from your general practitioner regarding my back-/leg-pain |  |
| e355 | Do you get sufficient support from other health professionals regarding my back-/leg-pain |  |
| e410 | Does you family's attitude and approach affect your pain/symptoms positively |  |
| e450 | Does the health professionals' attitude and approach affect your pain/symptoms positively |  |
| **14. Services, systems and policies** | | |
| e570 | Do you get the required support from the social services in relation to your needs, e.g. sickness benefit etc. | Not at all  Very little  Somewhat  Quite a lot  Very much |
| e580 | Are you satisfied with the available training- and treatment options you have been offered in relation to my back-/leg-pain |  |
| **15. Additional information** | | |
| - | If you have additional information about you and your pain / symptoms that is relevant for the healthcare professional , please state it here: | Open text box |

**Supplementary Material 2: Patient-Evaluation-Questionnaire (PEQ)**

| - **Item about use of PROs in the consultation** |  |
| --- | --- |
| To which degree was your responses from the PRO used in your dialogue with the health professional? | Not at all  Very little  Somewhat  Quite a lot  Very much |
| - **Item about presentation of the patient's profile in the consultation** |  |
| Did you see this report during the consultation? [a screenshot were presented to the patient] | Yes  No |
| - **The 9-items Shared Decision Making Questionnaire (SDM-Q-9)** | |
| - Please indicate which health complaint/problem/illness the consultation was about: | - Open text box |
| - Please indicate which decision was made: |  |
| - Nine statements related to the decision-making in your consultation are listed below. For each statement please indicate how much you agree or disagree. | |
| 1. My health professional made clear that a decision needs to be made | Completely disagree  Strongly disagree  Somewhat disagree  Somewhat agree  Strongly agree  Completely agree |
| 1. My health professional wanted to know exactly how I want to be involved in making the decision. |  |
| 1. My health professional told me that there are different options for treating my medical condition. |  |
| 1. My health professional precisely explained the advantages and disadvantages of the treatment options. |  |
| 1. My health professional helped me understand all the information. |  |
| 1. My health professional asked me which treatment option I prefer. |  |
| 1. My health professional and I thoroughly weighed the different treatment options |  |
| 1. My health professional and I selected a treatment option together. |  |
| 1. My do health professional and I reached an agreement on how to proceed |  |
